# Serological and viral genetic features of patients with COVID-19 in a selected German patient cohort—correlation with disease characteristics

**DOI:** 10.1007/s11357-021-00443-w

**Published:** 2021-09-01

**Authors:** Jonas Schmidt, Sandro Berghaus, Frithjof Blessing, Folker Wenzel, Holger Herbeck, Josef Blessing, Peter Schierack, Stefan Rödiger, Dirk Roggenbuck

**Affiliations:** 1 Institute for Laboratory Medicine, Singen, Germany; 2 Faculty of Medical and Life Sciences, Furtwangen University, Villingen-Schwenningen, Germany; 3 Faculty Environment and Natural Sciences, Institute of Biotechnology, Brandenburg University of Technology Cottbus-Senftenberg, Großenhainer Str. 57, 01968 Senftenberg, Germany; 4 Faculty of Health Sciences Brandenburg, Brandenburg University of Technology Cottbus – Senftenberg, Senftenberg, Germany

**Keywords:** COVID-19 disease characteristics, Serology, Viral genetics, Correlation

## Abstract

To study host-virus interactions after SARS coronavirus-2 (SARS-CoV-2) infection, genetic virus characteristics and the ensued humoral immune response were investigated for the first time. Fifty-five SARS-CoV-2-infected patients from the early pandemic phase were followed up including serological testing and whole genome sequencing. Anti-spike and nucleocapsid protein (S/N) IgG and IgM levels were determined by screening ELISA and IgG was further characterized by reactivity to S-subunit 1 (anti-S1), S-subunit 2 (anti-S2) and anti-N. In 55 patients, 90 genetic SARS-CoV-2 changes including 48 non-synonymous single nucleotide variants were identified. Phylogenetic analysis of the sequencing data showed a cluster representing a local outbreak and various family clusters. Anti-S/N and anti-N IgG were detected in 49 patients at an average of 83 days after blood collection. Anti-S/N IgM occurred significantly less frequently than IgG whereas anti-S2 was the least prevalent IgG reactivity (*P* < 0.05, respectively). Age and overweight were significantly associated with higher anti-S/N and anti-S1 IgG levels while age only with anti-N IgG (multiple regression, *P* < 0.05, respectively). Anti-S/N IgG/IgM levels, blood group A + , cardiovascular and tumour disease, NSP12 Q444H and ORF3a S177I were independent predictors of clinical characteristics with anti-S/N IgM being associated with the need for hospitalization (multivariate regression, *P* < 0.05, respectively). Anti-SARS-CoV-2 antibody generation was mainly affected by higher age and overweight in the present cohort. COVID-19 traits were associated with genetic SARS-CoV-2 variants, anti-S/N IgG/IgM levels, blood group A + and concomitant disease. Anti-S/N IgM was the only antibody associated with the need for hospitalization.

**Supplementary Information:**

The online version contains supplementary material available at 10.1007/s11357-021-00443-w.

## Introduction

Severe acute respiratory syndrome-coronavirus type 2 (SARS-CoV-2), an enveloped, positive-sense single-stranded RNA virus, causing coronavirus disease 2019 (COVID-19) has spread rapidly worldwide, with strong economic and social impacts [1, 2]. In contrast to endemic coronaviruses, SARS-CoV-2 is classified as highly pathogenic, with similar characteristics to SARS-CoV and Middle East respiratory syndrome (MERS)-CoV [3].

Its genome consists of 14 open-reading frames (ORF) [3, 4]. They encode 16 non-structural proteins (NSP) which are essential for virus replication within the host cell through the formation of a replicase complex [3, 4]. Additionally, the ORFs encode nine accessory and four structural proteins, which include spike (S), envelope, membrane and nucleocapsid (N) proteins [4]. Upon contact with the host cell, the S protein is cleaved into two subunits (S1/S2) by proteases [4]. Both of them are essential for viral entry and define tissue tropism as well as viral host range [4, 5].

After infection, the incubation period is approximately 4–12 days [4–6]. The clinical features of COVID-19 are diverse and vary in onset and severity [4]. Main symptoms are fever, cough, gastrointestinal illnesses, anosmia and dyspnoea [4]. In addition to these acute symptoms, COVID-19 may also be associated with long-term effects, such as myocardial inflammation [4]. In severe cases, initially mild symptoms may later progress to life-threatening systemic inflammation with a cytokine storm syndrome [1, 4]. This will result in acute respiratory distress syndrome and respiratory failure which are considered leading causes of death in patients with COVID-19 [1, 4].

Infection with SARS-CoV-2 triggers both humoral and cellular immune responses. However, the underlying molecular mechanisms are not fully understood [7]. The S and N proteins are most immunogenic, with distinct IgM, IgG and IgA responses noted in COVID-19 patients [7].

To study host-virus interactions, we combined clinical data of COVID-19 patients of a south-western region of Germany with comprehensive serological data and SARS-CoV-2 whole genome sequencing (WGS) results for the first time to our knowledge.

## Material and methods

### Study population

**Fig. 1:**
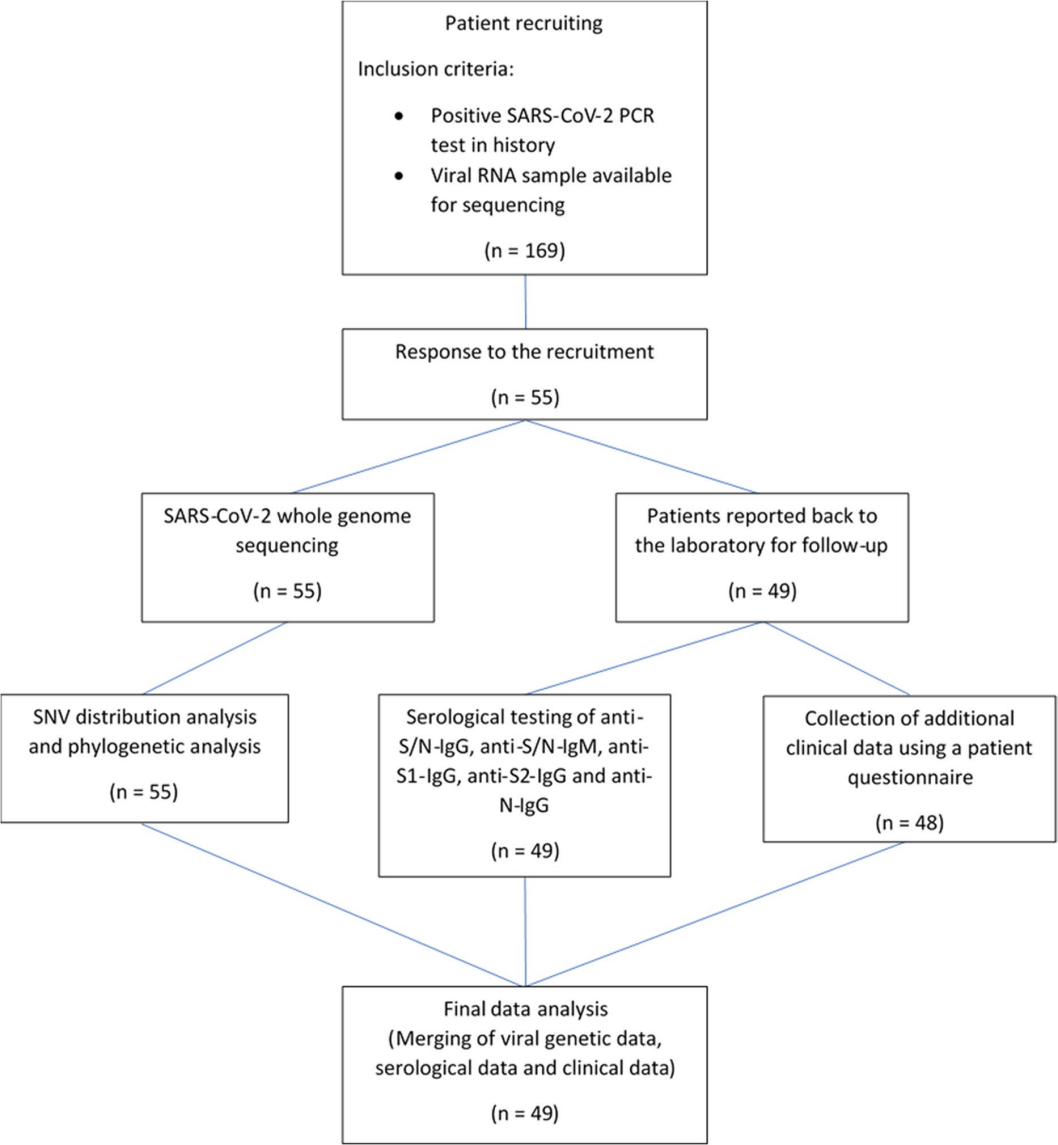
Recruitment of patients. Inclusion criteria for the study were a positive SARS-CoV-2 PCR test and a sample of the viral RNA present in the long-term sample archive. All patients were tested positive for SARS-CoV-2 in April 2020 at the beginning of the pandemic. anti-S/N, antibodies against a mixture of the spike glycoprotein with the nucleocapsid; anti-S1 IgG, IgG antibodies to spike glycoprotein domain 1; anti-S2 IgG, IgG antibodies to spike glycoprotein domain 2; anti-N IgG, IgG antibodies to nucleocapsid; SNV, single nucleotide variation

Fifty-five patients with COVID-19 diagnosed in accordance with the World Health Organization criteria from the State of Baden-Württemberg in Germany were included in this study. [8] Inclusion criteria were a positive SARS-CoV-2 PCR test and a sample of the viral RNA present in the long-term sample archive (Fig. 1).

In total, 169 individuals were tested positive for SARS-CoV-2 in April 2020 at the beginning of the pandemic. They were contacted at least 2 months later and were invited to participate in serological testing and clinical data collection from June to August. In six cases, a complete follow-up was not possible because the individual was deceased or not available for sample collection. The data collection by using a questionnaire included common patient data, risk factors, symptoms and duration of the disease, long-term effects, therapy and epidemiological questions (Table 1). Of the 49 patients, who underwent anti-SARS-CoV-2 antibody testing, 48 returned the questionnaire. Clinical progression was determined from the responses applying the proposed WHO clinical progression scale [9]. Need for hospitalization was reported by the study participants in the questionnaire.

**Table 1:** Patients’ and corresponding COVID-19 characteristics. In total, 48 returned a questionnaire, encompassing patient characteristics and clinical manifestations of the infection. One of the patients reported being completely symptom-free. The symptoms of the remaining 47 patients persisted for a median time of 10 days with an interquartile range of 7 days. Hospitalization due to moderate disease was reported in 6 cases with a mean hospitalization time of 7 days (standard deviation 5 days)

	Number/positive cases	Percentage [%]
Patient characteristics		
SARS-CoV-2 whole genome sequencing	55	100
Anti-SARS-CoV-2 antibody testing	49	89
Questionnaire complete	48	87
Death	5	9
Age		
< 30 years	5	9
30–65 years	36	65.4
> 65 years	14	25.5
BMI		
< 25^[a]^	17	35.4
25–35^[a]^	26	54.2
> 35^[a]^	5	10.4
Blood group^[b]^		
Type A +	16	33.4
Type A −	3	6.3
Type AB +	3	6.3
Type B +	3	6.3
Type O +	16	33.4
Type O −	2	4.2
Gender		
Female	29	52.7
Male	26	47.3
Clinical characteristics ^[a]^		
Cardiovascular disease	12	25
Chronic liver disease	2	4.2
Chronic lung disease	8	16.7
Diabetes	6	12.5
Tumour disease	3	6.3
Vitamin D supplementation	6	12.5
COVID-19 characteristics ^[a]^		
Appetite loss	29	60.4
Breathing difficulties	14	29.2
Bronchial secretions	12	25
Cough	26	54.2
Fatigue	43	89.5
Fever	27	56.3
Hospitalization	6	12.5
Without oxygen need	1	2.1
Oxygen need	5	10.4
Long-term COVID-19 effects	18	37.5
Night sweat	18	37.5
Pneumonia	4	8.3
Shortness of breath	9	18.8
Sore throat	17	35.4
Taste and smell disorders	32	66.7

^[a]^Only patients with a complete questionnaire are included (*n* = 48)

^[b]^Blood groups were only available from 43 patients

### Serological testing

Serum samples for serological testing were collected by venipuncture and stored at − 20 °C until further analysis. The anti-SARS-CoV-2 IgG and IgM levels to a mixture of S and N proteins (anti-S/N), respectively, were determined according to the manufacturer’s manual by two commercial ELISA kits (GA CoV-2 IgG, GA CoV-2 IgM, GA Generic Assays GmbH, Dahlewitz, Germany) on an automated ELISA analyser (Institut Viron-Serion GmbH, Würzburg, Germany). Briefly, a binding index (BI) is calculated by the ratio of optical density (OD) values of samples to a cut-off OD value. Results with a BI ranging from 0.9 to 1.1 were considered borderline [10].

Additionally, an anti-SARS-CoV-2 IgG ELISA, recommended for confirmatory anti-SARS-CoV-2 IgG testing, was performed according to the manufacturer’s protocol (GA CoV-2 IgG + , GA Generic Assays). The assay differentiates IgG to S1 (anti-S1), S2 (anti-S2) and N proteins (anti-N).

All antibody assays showed sensitivities of ≥ 98% after 14 days of SARS-CoV-2 confirmation by PCR. To assess specificity, 1000 blood donor samples collected before and after the COVID-19 outbreak were tested. The anti-S/N IgG and IgM assays showed a specificity of > 98%, respectively. False-positive results may be a consequence of the previous contact with other members of the coronavirus family. No cross-reactions were found by antibodies to the following common infective agents: PIV1-3, Influenza viruses A and B, Haemophilus influenzae, hCoV-229E, hCoV-OC43, hCoV-HKU1, hCoV-NL63, rhinovirus, RSV, adenovirus, M. pneumoniae, C. pneumoniae, CMV, EBV, HSV1 and 2, Toxoplasma, Rubella virus, Coxsackie virus, Parvovirus B19, HCV and HIV. The detected false-positive antibodies were mainly reactive with the N protein. These antibodies were probably generated during previous infections by endemic coronaviruses. Using samples first tested negative for IgG on the GA CoV-2 IgG ELISA, the GA CoV-2 IgG + reached a specificity of almost 100%.

### PCR testing

Viral RNA was isolated from nasopharyngeal swaps using PrepitoViral DNA/RNA300 isolation kits (PerkinElmer, Waltham, USA). PCR testing was performed by using the QuantiTect Probe RT-PCR Kit (Qiagen, Hilden, Germany) with primers and a hydrolysis probe (Biomers, Ulm, Germany) targeting the E gene (Suppl. Material 1). Detection was done on the FAM channel of a LightCycler 96 instrument (Roche, Basel, Switzerland).

### SARS-CoV-2 next-generation sequencing

SARS-CoV-2 WGS was performed on a MinION sequencing platform (Oxford Nanopore Technologies, Oxford, UK) using the ARTIC nCoV-2019 sequencing protocol (Suppl. Mat. 2) [11–13]. All 55 samples were divided into three sequencing runs, each including a no-template control and an internal sequencing control. Lambda DNA (Oxford Nanopore Technologies, Oxford, UK) was used as an internal control.

### Sequencing data analysis

Rampart was used to monitor the sequencing runs in real time. Oxford Nanopores own basecaller Guppy was employed to rebasecall the produced FAST5 files with a high accuracy model and for demultiplexing. Detailed analysis of sequence data is outlined in Supplemental Material 2. The resulting phylogenetic tree was visualized using R (v4.0.2) (R Foundation for Statistical Computing, Vienna, Austria) and the ggtree package (Suppl. Tab. 1). All consensus sequences from this study are available from GISAID (Suppl. Material 2).

### Statistical analysis

Statistical testing was performed using R and ggplot2 package as well as MedCalc (v13.3.00) (MedCalc Software Ltd., Ostend, Belgium). Normality of data was assessed by Shapiro–Wilk test. In the case data was not normally distributed, differences between patient groups were compared using Kruskal–Wallis tests followed by post hoc analysis according to Conover. To compare the variation rate of different genes in the SARS-CoV-2 genome relative to their length, a generalized linear model (GLM) assuming a Poisson distribution was applied. Rank correlation was performed to identify the degree of association between antibody levels and patient characteristics. Logistic regression and multiple regression analyses were performed to predict an association between clinical outcome, serological data and genetic SARS-CoV-2 characteristics.

## Results

### Clinical presentation of COVID-19

To gain a deeper understanding of SARS-CoV-2 host-virus interactions, a follow-up of 55 COVID-19 patients from April 2020 was performed encompassing (i) SARS-CoV-2 WGS and (ii) serological testing for anti-S/N IgG and IgM as well as IgG to S1, S2 and N. Of 55 COVID-19 patients with PCR-confirmed SARS-CoV-2 infection and viral WGS analysis, 49 patients reported back to the laboratory for antibody testing (Fig. 1). In five of the 6 cases without follow-up, the patient was deceased. Of these 49 patients with a mean age of 52.2 years (standard deviation [SD] 16.2 years), 48 returned a questionnaire, encompassing patient characteristics and clinical manifestations of the infection (Table 1). One of the patients reported being completely symptom-free. The symptoms of the remaining 47 patients persisted for a median time of 10 days (interquartile range [IQR] 7 days). Hospitalization due to moderate disease was reported in 6 cases with a mean hospitalization time of 7 days (SD 5.0 days). Long-term effects of COVID-19 were stated by 18 patients (37.5%), including primarily fatigue and persisting loss of taste and smell.

### SARS-CoV-2 whole genome sequencing

**Fig. 2:**
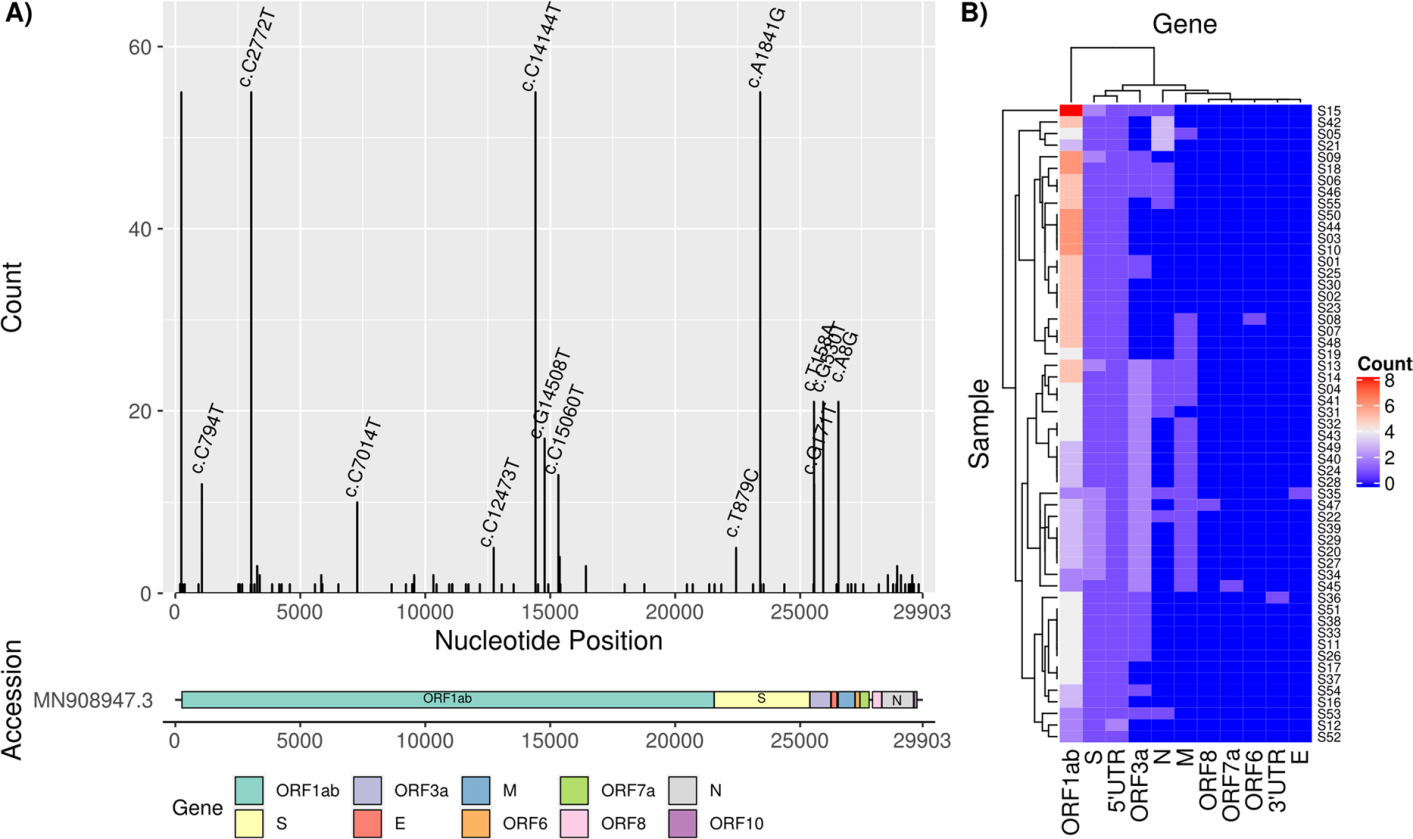
**A** Overall distribution of SARS-CoV-2 variants identified by whole genome sequencing. The most common variants in coding regions are labelled. **B** Individual variant count per gene in each study sample

Whole genome sequencing of 55 SARS-CoV-2 RNA samples of the recruited COVID-19 patients was performed whereas all obtained sequences could be included in further downstream analysis as the coverage was above 85% (min 88.9%; max 99.6%). Variants to the reference genome MN908947.3 were clearly distributed over the whole SARS-CoV-2 genome (Fig. 2A). In total, 90 different unique variants including 34 synonymous single nucleotide variations (SNVs), 48 non-synonymous SNVs, 2 non-frameshift insertions, 1 frameshift insertion and 5 unclassified variants were identified within the study population (Suppl. Tab. 2). Median variant count per sample was eight and 99.7% of the genomic sites in the total population were without variations. The variants c.C2772T (ORF1ab F924F), c.C14144T (ORF1ab P4715L), c.A1841G (S D614G), and a transition from C to T in the 5ʹ UTR at position 241 were identified in all 55 samples (Fig. 2A). A heat map of the variant count per gene and sample demonstrated that ORF10 was the only invariant region (Fig. 2B). In all samples, the highest numbers of variants were found in ORF1ab, followed by S, 5ʹ UTR and ORF3a.

The variation rate of the individual genes relative to their length was assessed by a general linearized model (Suppl. Figure 1). Here, a highly significant positive influence of the N gene on the normalized variation rate was identified (*P* = 0.0096, estimate: 0.876, standard error [SE]: 0.338), which means that this gene shows a significantly larger number of unique variants compared to the other regions of the SARS-CoV-2 genome. Further to this, a significant negative influence of ORF1ab on the normalized variation rate was observed by applying the model (*P* = 0.04, estimate: − 0.528, SE: 0.258).

**Fig. 3:**
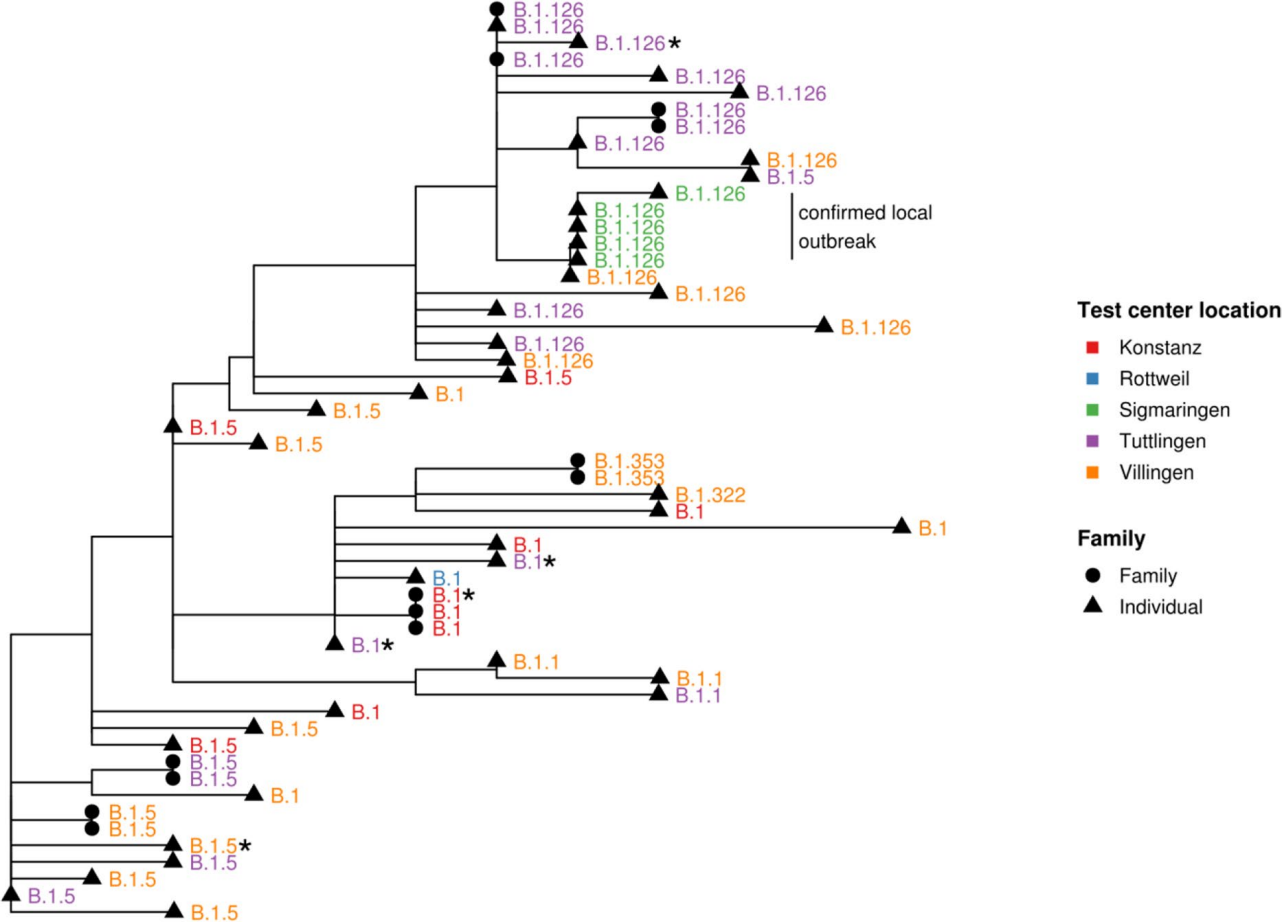
Phylogenetic analysis of the different SARS-CoV-2 consensus sequences. The tips of the tree are labelled with the identified lineage (* = deceased patients). Samples from patients, which belong to the same family, present clear clusters. Additionally, a local outbreak in the region of Sigmaringen with the lineage B.1.126 was identified

To analyse the sequencing data from an epidemiological perspective, a phylogenetic analysis was performed (Fig. 3). Six different SARS-CoV-2 lineages, namely B.1, B.1.1, B.1.5, B.1.126, B.1.322 and B.1.353 were identified (Suppl. Tab. 3). The phylogenetic tree showed clear regional clusters in the area of Tuttlingen and Sigmaringen. Deeper analysis of patients’ meta-data from the questionnaire revealed that the local cluster in the area of Sigmaringen originated from a local outbreak in a rehabilitation clinic. This was also confirmed by local health authorities. Besides local clustering, distinct clusters were observed within family members all of whom had an identical SARS-CoV-2 genotype.

### Serological testing

**Fig. 4:**
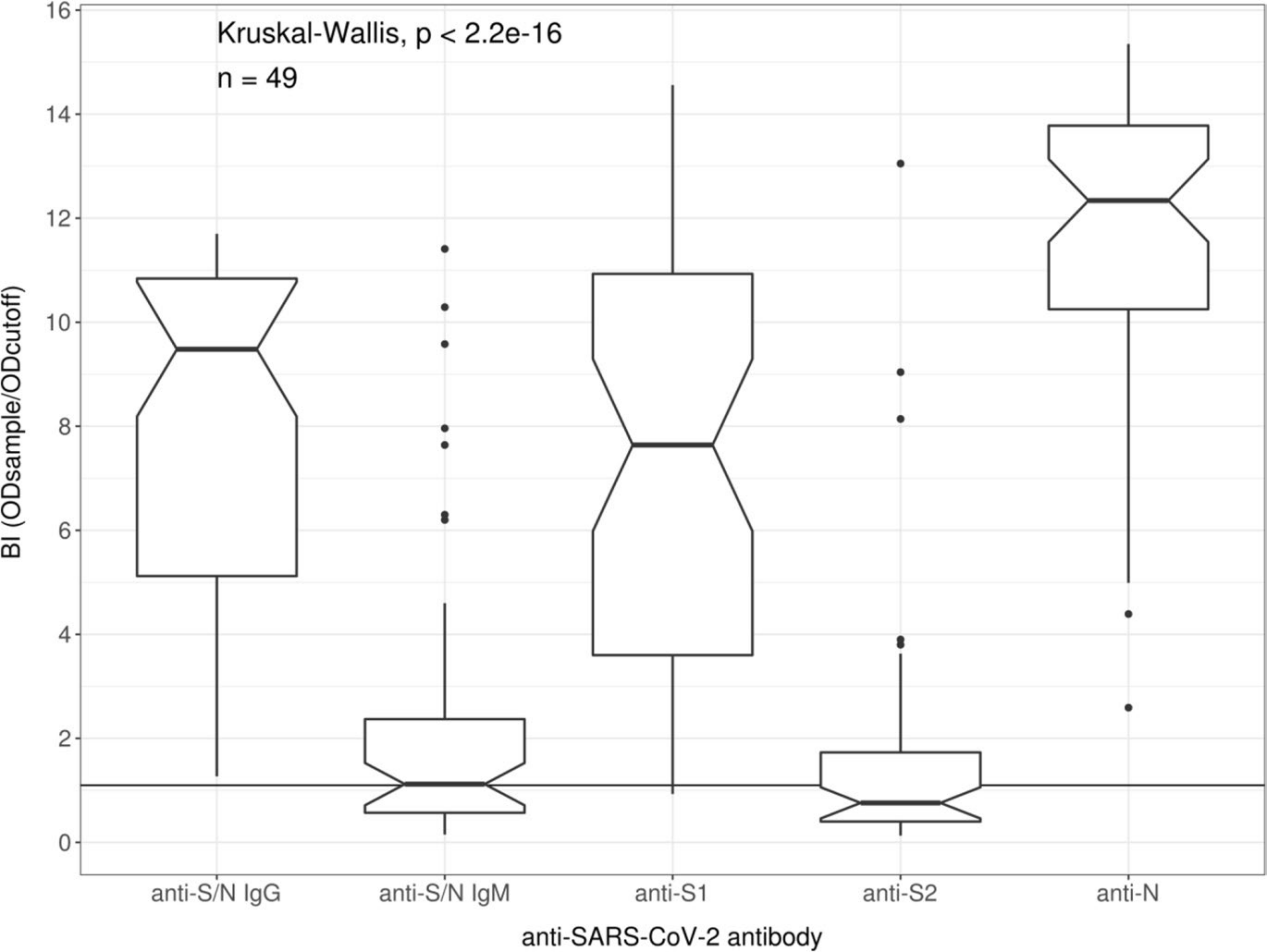
Anti-SARS-CoV-2 antibody levels in 49 patients with COVID-19. In total, 49 patient samples were tested for anti-SARS-CoV-2 IgG and IgM against a mixture of the spike glycoprotein with the nucleocapsid (anti-S/N), respectively. Furthermore, IgG against the spike glycoprotein domain 1 (anti-S1), domain 2 (anti-S2) and the nucleocapsid protein (anti-N) were detected. The positive cut-off is located at 1.1 BI (binding index)

Blood drawing was performed on average 83 days (mean 83.3 days, SD 14.3 days) after a positive PCR result. Serological testing encompassed the semiquantitative detection of anti-S/N IgG and IgM levels. Additionally, IgG levels were differentiated into anti-S1, anti-S2 and anti-N IgG (Fig. 4).

Anti-S/N IgG and anti-N IgG were detected in all 49 patients. Anti-S/N IgM was less frequently detected than anti-S/N IgG (27/49 vs. 49/49, *P* < 0.0001). Among the three IgG reactivities investigated, anti-S2 IgG occurred significantly less frequently than anti-S1 and anti-N IgG (19/49 vs. 48/49 and 49/49, *P* < 0.0001, respectively).

Patients demonstrated antibody patterns with varying frequencies (Table 2). The three most prevalent patterns (anti-S/N, anti-S1 and anti-N IgG; anti-S/N IgG and IgM, anti-S1 and anti-N IgG; anti-S/N IgG and IgM, anti-S1, anti-S2 and anti-N IgG) did not show a significantly different prevalence (*P* > 0.05, respectively).

**Table 2:** Patterns of anti-SARS-CoV-2 antibody positivity by ELISA. In total 49 patient samples were tested for anti-SARS-CoV-2 IgG and IgM against a mixture of the spike glycoprotein with the nucleocapsid (anti-S/N), respectively. Furthermore, IgG against the spike glycoprotein domain 1 (anti-S1), domain 2 (anti-S2) and the nucleocapsid protein (anti-N) were detected. Five different anti-SARS-CoV-2 antibody patterns were identified by ELISA testing. Patterns I, II and III were significantly more prevalent than patterns IV and V (Fisher’s exact test, *P* < 0.05, respectively)

Pattern	Anti-S/N IgG	Anti-S/N IgM	Anti-S1 IgG	Anti-S2 IgG	Anti-N IgG	Number	Percentage [%]
I	+	−	+	−	+	15	30.6
II	+	+	+	−	+	15	30.6
III	+	+	+	+	+	12	24.5
IV	+	−	+	+	+	6	12.2
V	+	−	−	+	+	1	2.0

+  = positive, −  = negative

The obtained IgG and IgM levels did not correlate within the examined period of 83 days on average after SARS-CoV-2 PCR testing (*P* > 0.05, respectively).

#### Anti-SARS-CoV-2 antibody levels in age groups

Rank correlation analysis revealed significant associations of all anti-SARS-CoV-2 antibodies with age (anti-S/N IgG, Spearman’s rho [*ϕ*] = 0.497, *P* = 0.0003; anti-S/N IgM, *ϕ* = 0.312, *P* = 0.0289; anti-N IgG, *ϕ* = 0.485, *P* = 0.0004; anti-S1 IgG, *ϕ* = 0.521, *P* = 0.0001; anti-S2 IgG, *ϕ* = 0.288, *P* = 0.0451).

**Fig. 5:**
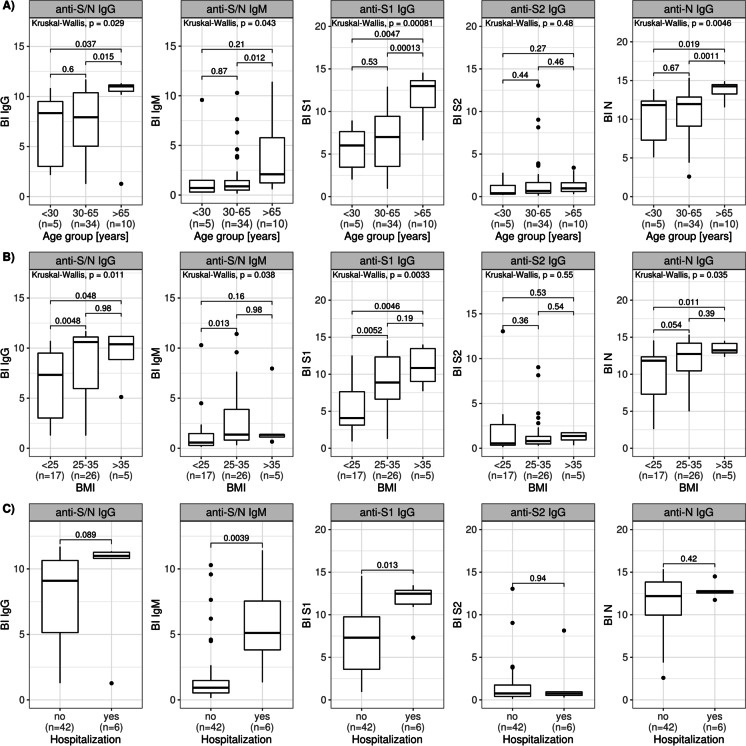
Anti-SARS-CoV-2 antibody levels differentiated by age, BMI and severity. **A** Anti-SARS-CoV-2 antibody levels in different age groups (*n* = 49). **B** Anti-SARS-CoV-2 antibody levels in different body mass index (BMI) groups (*n* = 48). **C** Anti-SARS-CoV-2 antibody levels in relation to the need for hospitalization (*n* = 48)

To further investigate the occurrence of anti-SARS-CoV-2 antibodies in relation to age, patients were stratified into three groups: (i) younger than 30 years (*n* = 5), (ii) between 30 and 65 years (*n* = 34), and (iii) older than 65 years (*n* = 10). Patients older than 65 years showed significantly higher anti-S/N, anti-S1 and anti-N IgG levels in contrast to patients in the two groups with younger age (*P* < 0.05 respectively) (Fig. 5A). Anti-S/N IgM levels were significantly higher only in patients older than 65 years compared to patients aged 30–65 years (*P* = 0.012), but not compared to the age group below 30 years (*P* > 0.05). For anti-S2 IgG, no significant differences between the age groups were observed.

#### Anti-SARS-CoV-2 antibody levels in groups with different BMI

The body mass index (BMI) was calculated and correlated with the various anti-SARS-CoV-2 antibodies. A significant association was established for anti-S/N IgG (*ϕ* = 0.404, *P* = 0.0045), anti-S/N IgM (*ϕ* = 0.355, *P* = 0.0133) and anti-S1 IgG (*ϕ* = 0.451, *P* = 0.0013).

Furthermore, patients were stratified into three different groups: (i) normal weight (BMI < 25; *n* = 17), (ii) overweight (BMI 25–35; *n* = 26), (iii) severe overweight (BMI > 35, *n* = 5). Patients with overweight and severe overweight showed significantly higher antibody levels compared to the normal weight group for all tested antibodies except anti-S/N IgM and anti-S2 IgG (*P* < 0.05, respectively) (Fig. 5B). Anti-S/N IgM levels were only significantly higher in patients of the overweight group compared with the ones of the normal weight group (*P* = 0.013).

#### Anti-SARS-CoV-2 antibody levels in relation to the need for hospitalization

Furthermore, antibody levels were compared with regard to the need for hospitalization indicating moderate COVID19 with scores ranging from 4 to 5 (Fig. 5C). Here, significantly higher levels of anti-S/N IgM and anti-S1 IgG were observed in hospitalized patients (*n* = 6, *P* < 0.05, respectively). All other antibodies tested demonstrated no significant difference regarding the need for hospitalization (*P* > 0.05, respectively).

A possible association of anti-SARS-CoV-2 antibodies with hospitalization duration was investigated by rank correlation. Again, a significant association was observed for anti-S/N IgM (*ϕ* = 0.428, *P* = 0.0024) and anti-S1 IgG (*ϕ* = 0.355, *P* = 0.0133).

#### Association of anti-SARS-CoV-2 antibody levels with genetic SARS-CoV-2 variants and patient characteristics

Given the positive correlation of anti-SARS-CoV-2 antibody levels with age and overweight, univariate followed by multivariate regression analysis was performed to investigate an influence of other patient characteristics and genetic SARS-CoV-2 variants on antibody generation (Table 3). Age was established as an independent predictor for higher anti-S/N, anti-S1 and anti-N IgG levels whereas the latter had no further predictors. In contrast, overweight (BMI > 25, *n* = 31) was identified as an additional independent predictor for higher anti-S/N and anti-S1 IgG levels. The absence of the genetic SARS-CoV-2 variant NSP3 D218E was an additional independent predictor for higher anti-S1 IgG levels whereas the absence of chronic liver disease was one for higher anti-S/N IgG levels.

**Table 3:** Multiple regression analyses of anti-SARS-CoV-2 antibody levels of 48 patients as dependent variables and independent parameters encompassing patient characteristics listed in Table 1 and SARS-CoV-2 genetic features as predictors. *anti-S/N IgG*, IgG antibodies against a mixture of the spike glycoprotein with the nucleocapsid; *anti-S1 IgG*, IgG antibodies to spike glycoprotein domain 1; *anti-N IgG*, IgG antibodies to nucleocapsid

	Coefficient	Std. Error	*P* value
Anti-S/N IgG			
Age	0.069	0.026	0.0104
Chronic liver disease	− 5.225	2.020	0.0131
Overweight ^[a]^	2.163	0.876	0.0174
Anti-S/N IgM			
Tumour disease	5.064	1.567	0.0023
Anti-S1 IgG			
Age	0.088	0.030	0.0049
Overweight ^[a]^	2.828	1.005	0.0073
NSP3 D218E	− 5.708	2.313	0.0175
Anti-S2 IgG			
NSP3 D218E	4.837	1.662	0.0055
Anti-N IgG			
Age	0.081	0.026	0.0033

^[a]^Overweight was characterized by BMI > 25

The only independent predictor for higher anti-S/N IgM levels was the presence of tumour disease with no predictive effect of genetic SARS-CoV-2 variants or other patient characteristics such as age and overweight. For higher anti-S2 IgG levels, the presence of NSP3 D218E was revealed as the only independent predictor, which is in strong contrast to anti-S1 IgG.

### Association between clinical outcome, genetic SARS-CoV-2 variability, humoral immune response and patient characteristics

In light of the correlation of anti-SARS-CoV-2 antibody levels with the need for hospitalization and its duration, univariate followed by multivariate regression analyses were performed to evaluate a possible association between the clinical outcome and various independent predictor variables (patient characteristics, antibody levels, viral genetic features).

Univariate analysis revealed a number of clinical characteristics as the dependent variable, which had higher SARS-CoV-2-antibody levels other than anti-S2 IgG levels as independent predictors (Suppl. Tab. 4). A total of five SNVs were found to be independent predictors of COVID-19 traits. All of them were non-synonymous, resulting in amino acid changes in various viral proteins.

In subsequent multivariate logistic regression analysis to account for confounding variables, only higher anti-S/N IgG and/or IgM levels were found to significantly predict COVID-19 characteristics such as appetite loss, night sweat, oxygen need, pneumonia and the need for hospitalization (*P* < 0.05, respectively) (Table 4). Interestingly, anti-S/N IgM was the only variable studied that predicted the occurrence of pneumonia (odds ratio [OR] 1.363, *P* = 0.0317). Furthermore, the main confounder for higher anti-S/N IgM levels was cardiovascular disease in the prediction of the need for oxygen and hospitalization (*P* < 0.05, respectively). The blood group A + was identified as an independent predictor for bronchial secretions and cough whereas the latter demonstrated the SNV ORF3a S177I as an additional independent predictor (*P* < 0.05, respectively). The only other SNV identified as independent was NSP12 Q444H for taste and smell disorders (OR 5.444, *P* = 0.0426).

**Table 4:** Multivariate regression analyses of COVID-19 characteristics of 48 patients. (A) Multivariate regression analyses of binary COVID-19 patient characteristics by logistic regression analysis. The relationship of dichotomous COVID-19 patient characteristics as dependent variables and independent parameters encompassing patient characteristics listed in Table 1, SARS-CoV-2 genetic features and anti-SARS-CoV-2 antibodies as predictors was analysed. (B) Multiple regression analyses of quantitative COVID-19 patient characteristics as dependent variables and independent parameters encompassing patient characteristics listed in Table 1, SARS-CoV-2 genetic features and anti-SARS-CoV-2 antibodies as predictors

(A) Logistic regression	Coefficient	Std. error	Odds ratio	95% CI	*P* value
Appetite loss					
Anti-S/N IgG	0.367	0.114	1.443	1.155–1.802	0.0012
Bronchial secretions					
Blood type A +	1.749	0.737	5.750	1.356–24.389	0.0177
Cough					
Blood type A +	2.765	1.144	15.882	1.687–149.490	0.0156
ORF3a S177I	− 3.041	1.108	0.048	0.054–0.419	0.0061
Night sweat					
Anti-S/N IgG	0.404	0.153	1.498	1.109–2.023	0.0084
Anti-S/N IgM	0.300	0.148	1.350	1.011–1.804	0.0419
Oxygen need					
Anti-S/N IgM	0.413	0.188	1.511	1.045–2.185	0.0282
Cardiovascular disease	3.075	1.432	21.647	1.306–358.738	0.0318
Pneumonia					
Anti-S/N IgM	0.310	0.144	1.363	1.027–1.808	0.0317
Hospitalization					
Anti-S/N IgM	0.441	0.201	1.554	1.992–2.306	0.0284
Cardiovascular disease	3.708	1.540	40.773	1.992–834.549	0.0161
Taste and smell disorders					
NSP12 Q444H	1.695	0.836	5.444	1.058–28.011	0.0426
** (B) Multiple regression**	**Coefficient**		**Std. error**		***P* value**
Hospitalization duration					
Anti-S/N IgM	0.305		0.109		0.0075
Tumour disease	5.980		1.300		< 0.0001
Chronical lung disease	2.224		3.334		0.0017
Symptom duration					
Chronical lung disease	19.250		7.456		0.0053
N E253A	12.571		4.261		0.0137

Along with the presence of tumour and chronic lung diseases, a higher anti-S/N IgM level was significantly associated with longer hospitalization (multiple regression analysis, *P* < 0.05, respectively).

Chronic lung disease and the SNV N E253A were significantly associated with symptom duration (multiple regression analysis, *P* < 0.05, respectively).

## Discussion

More than a year after its identification, SARS-CoV-2 has shown a high degree of genome alteration [14]. To investigate virus-host interactions, we examined PCR-positive patients of a south-western German region who were referred to a local reference laboratory and answered a questionnaire on personal and COVID-19 characteristics.

Thus, WGS of the viral genome of 55 enrolled COVID-19 patient samples revealed genetic alterations mainly as SNVs, with about half of these resulting in changes of the amino acid sequence. When looking at the absolute variant count per gene and patient, most variants were located within ORF1ab representing the largest SARS-CoV-2 ORF. Nevertheless, ORF1ab showed a significantly lower variation rate normalized on the gene length compared to the other genes, while the N gene was the only gene with a significantly higher normalized variation rate. Overall, RNA viruses are known to accumulate variants rapidly during their replication cycle because RNA copying enzymes are prone to error [15, 16]. A high variation rate of the N gene was reported elsewhere [17, 18].

ORF10 was the only gene without variants in our study which was also demonstrated elsewhere [18]. Furthermore, our study corroborated published data on the S gene stability [19].

We observed four variants present in all samples (ORF1ab F924F, ORF1ab P4715L, S D614G and 5ʹUTR 241C > T), representing signature variants of the most dominant SARS-CoV-2 type VI strain [20]. In particular, the D614G exchange in the S protein has been extensively studied and is postulated to provide a selection advantage through increased viral infectivity [21–23].

All samples were assigned to the root lineage B based on Rambaut’s nomenclature [24]. The highest level lineage was B.1, encompassing the major Italian outbreak in early 2020 and then spreading across Europe [24]. The other identified lineages were sub-lineages of B.1, which match the geographical origin of the samples. Remarkably, the earliest description dates of the lineages in the Pango strain database coincided with our sample collection date (2020–04-07 to 2020–05-07). At the time of writing this manuscript, the lineages B.1.322, B.1.353 and B.1.5 have already been reassigned as more and more SARS-CoV-2 whole genomes have been sequenced over time and lineage formation and extinction continue to progress [24].

Given the high genetic variability of SARS-CoV-2, we sought to investigate the emergence of the humoral immune response by determining specific IgM and IgG against the most immunogenic S and N proteins in average 83 days after PCR testing [25, 26]. As expected, all patients revealed detectable anti-S/N and anti-N IgG while only one patient out of the examined 49 did not show anti-S1 IgG. The higher anti-S/N IgG prevalence in contrast to IgM probably indicates the effect of an immunological memory likely induced by previous infections with endemic coronaviruses, as primary immune responses would induce stronger anti-SARS-CoV-2 IgM responses. For all antibodies tested, there was no correlation between time from SARS-CoV-2 PCR testing and antibody levels within the examined period of 83 days on average after SARS-CoV-2 PCR testing. However, it cannot be ruled out that anti-S/N IgM levels, in particular, may have decreased to negative values in the period leading up to blood collection for antibody determination.

Rank correlation and multiple regression analyses using genetic SARS-CoV-2 variants and patient characteristics as independent variables for the prediction of anti-SARS-CoV-2 antibody levels revealed an association of older age (> 65 years) and overweight (BMI > 25) with higher anti-S/N and anti-S1 IgG levels. In contrast, higher anti-N IgG levels were only associated with older age. The average age of enrolled patients was 52.2 years which is in agreement with the reported age of around 50 years for COVID-19 patients [1, 27]. A systematic review and meta-analysis found old age and obesity as a risk for a severe COVID-19 course [28].

Remarkably, despite a positive correlation of age and BMI with anti-S/N IgM, higher levels of the latter were only associated with the concurrence of tumour disease by multiple regression analysis. On the contrary, the absence of concomitant chronic liver disease was a confounder for the association of older age and overweight with higher IgG levels. The found correlation with older age reflects the stronger humoral inflammatory response reported in aged COVID-19 patients, which may hint at an impaired innate or cellular adaptive immune response [1, 29]. Apart from older age, overweight has been described as an additional risk factor for severe COVID-19 progression usually linked with functional impairment of immune cells and decreased immunity as a result of chronic inflammation and hypercytokinemia [30, 31]. Therefore, the observed positive association with higher anti-S/N and anti-S1 IgG levels may also be due to a unique predisposition of obese individuals to an impaired cellular anti-SARS-CoV-2 response and requires further investigation. Significantly higher SARS-CoV-2 IgG levels were also previously described in patients with metabolic syndrome comorbidities [32].

In line with previous reports, higher anti-S1 IgG levels were determined in contrast to anti-S2 IgG levels [26]. For the first time, we showed the positive association of higher anti-S2 IgG levels with the SNV NSP3 D218E. This is interesting as the same SNV is negatively associated with higher anti-S1 IgG levels in our patient cohort and may indicate a possible influence of SARS-CoV-2 non-structural protein 3 (NSP3) on antibody formation. The multi-domain Nsp3 is the largest SARS-CoV-2 protein and an essential component of the replication-transcription complex modifying host proteins and interfering with innate immune responses by de-ubiquitination [33].

There was an association of higher anti-S/N IgM and anti-S1 IgG levels with moderate COVID-19 requiring hospitalization of patients. Both anti-SARS-CoV-2 antibodies were also positively correlated with hospitalization duration. Multivariate regression analysis identified only higher anti-S/N IgM levels as predictors for the need for hospitalization with concomitant cardiovascular disease as confounder. This could entail that anti-S/N IgM can be employed as a marker of at least moderate COVID-19 in particular for patients with cardiovascular disease. Cardiovascular disease is an accepted risk factor for severe COVID-19 courses [34, 35].

In light of the diverse clinical expression of COVID-19 in our study cohort, the varying predisposition of patients and the genetic changes of SARS-CoV-2, we performed univariate followed by multivariate regression analysis to identify possible associations. COVID-19 symptoms observed in our study cohort were consistent with other studies [1, 27].

Interestingly, higher anti-S/N IgM and IgG levels were established as independent predictors of COVID-19 traits such as appetite loss, night sweat, oxygen need and pneumonia. The latter was associated only with higher anti-S/N IgM levels without confounders, supporting published data and the above correlation of the IgM response with the need for hospitalization [36]. In addition to the presence of tumour and chronic lung disease, hospitalization duration was also associated with higher anti-S/N IgM levels.

Another interesting association was the prediction of clinical symptoms such as cough and bronchial secretions by blood type A + . This is consistent with other studies demonstrating a higher risk of individuals with this blood type to develop COVID-19 symptoms after infection [37–39]. While the occurrence of bronchial secretions was only associated with blood type A, the absence of the non-synonymous SNV ORF3a S177I was a confounder for the appearance of cough. The prediction of taste and smell disorders by the non-synonymous SNV NSP12 Q444H (OR 5.4) without confounders is another example in this study that genetic changes may influence the clinical presentation of COVID-19 [22, 40–44]. NSP12 is a large SARS-CoV-2 protein with 932 amino acid residues catalysing replication and transcription of the viral genome [45]. Furthermore, patients with chronic lung disease infected with SARS-CoV-2 bearing the non-synonymous SNV N E253A appear to have a longer symptom duration. This N protein SNV was the only genetic change in structural proteins associated with clinical characteristics in this study. The N protein demonstrating a high level of genetic alteration in the study has multiple functions including complex formation with genomic RNA, interaction with the viral membrane protein during virion assembly and enhancement of the efficiency of virus transcription and assembly [46]. However, it is not part of the replication-transcription complex which is the core component during viral replication [4, 5].

Approximately one-third of patients (*n* = 18) in our study population reported having long-term symptoms, particularly persistent anosmia and fatigue after recovery from COVID-19. We could not find statistically significant associations with the persistency of symptoms.

A limitation of our study is the relatively small sample size. In addition, data may be biased by preferential inclusion of patients with symptoms. There was only one patient that did not report COVID-19 symptoms. Therefore, confirmation of the findings in a larger study population is warranted. Additionally, the associations identified between certain viral and patient characteristics and the clinical outcome of COVID-19 are only descriptive. However, this is the first study combining SARS-CoV-2 WGS with comprehensive anti-SARS-CoV-2 antibody testing encompassing IgM and IgG reactivities.

## Conclusion

Our results show diverse humoral immune responses to SARS-CoV-2, which appear to be influenced by disease severity, age and obesity. The serologic profile is more like that of a secondary humoral immune response than a primary one. The non-synonymous SARS-CoV-2 SNV NSP3 D218E is inversely associated with the humoral response to S subunits 1 and 2.

Clinical COVID-19 characteristics are correlated with genetic changes of SARS-CoV-2, anti-S/N IgG and IgM levels as well as patient characteristics such as blood type A + . Anti-S/N IgM is correlated with pneumonia and the need for hospitalization and oxygen. We identified the N gene to be the most variable part of the SARS-CoV-2 genome.

## Supplementary Information

Below is the link to the electronic supplementary material.

Supplementary file1 (DOCX 143 KB)

## Data Availability

The data sets are available in aggregated form on request from the authors.

## Code availability

Custom R scripts are available on request from the authors.
