# Supplementary material for: Serological and viral genetic features of patients with COVID-19 in a selected German patient cohort—correlation with disease characteristics
Source: GeroScience. 2021 Sep 1;43(5):2249–64. doi: 10.1007/s11357-021-00443-w (PMC8408040; doi:10.1007/s11357-021-00443-w)
Supplement: Supplementary file 1 — Supplementary file1 (DOCX 143 KB) [file 11357_2021_443_MOESM1_ESM.docx]

**Supplemental Material 1:**

RT-PCR primers, probe and temperature profile used for SARS-CoV-2 detection.

SARS-CoV-2 E gene PCR Primers (5’ - 3’):

Forward: ACAGGTACGTTAATAGTTAATAGCG
Reverse: TATTGCAGCAGTACGCACAC

SARS-CoV-2 E gene hydrolysis probe (5’ FAM - 3’ BBQ):

ACACTAGCCATCCTTACTGCGCTTCG

SARS-CoV-2 RT-PCR temperature profile:

| Step | Time [min] | Temperature [°C] | Cycle |
| --- | --- | --- | --- |
| Reverse Transcription | 10:00 | 55 | - |
| Initial Denaturation | 3:00 | 94 | - |
| Denaturation | 00:15 | 94 | 45 |
| Annealing | 00:30 | 58 |  |

**Supplemental Material 2:**

SARS-CoV-2 next generation sequencing and analysis

After PCR testing, positive isolates were stored at -70 °C until all corresponding serum samples were collected. According to the ARTIC protocol prior to sequencing, a reverse transcription followed by a multiplex PCR using the ARTIC nCoV-2019 V3 primer set (Integrated DNA Technologies, Coralville, USA) were performed. Successful amplification was tested with conventional gel electrophoresis and Qubit measurements (Thermo Fisher Scientific, Waltham, USA). During the following sequencing library preparation, the samples were barcoded by using native barcoding with 24 different barcodes (Oxford Nanopore Technologies, Oxford, United Kingdom). Sequencing on the MinION was performed for 12h on a R9.4.1 flow cell (Oxford Nanopore Technologies, Oxford, United Kingdom). Overall raw data quality was assessed by applying pycoQC. Briefly, reads were filtered for a length between 400 and 700 nucleotides and a minium quality score of 12 using guppyplex from the ARTIC pipeline to exclude chimeric and low-quality reads. The filtered reads were used for consensus-sequence generation and variant calling again using the ARTIC pipeline. Consensus-sequence quality control was done with a custom R script determining coverage, depth and sequence identity to the target genome. Finally, lineage classification of the individual sequences was performed using Pangolin. Visualization and analysis of the variant distribution was performed by ANNOVAR and custom R scripts (gggenes, ggpubr, ggplot2, ComplexHeatmap). Phylogenetic analysis was done by MAFFT for iterative refinement (L-INS-i) multiple sequence alignment and PHyML to analyse the alignments in a phylogenetic framework using Maximum-Likelihood Phylogenies. A HKY85 model with gamma distribution was set.

GISAID IDs of SARS-CoV-2 whole genome sequences generated in this study:

EPI_ISL_640259, EPI_ISL_640258, EPI_ISL_640257, EPI_ISL_640219, EPI_ISL_640263, EPI_ISL_640262, EPI_ISL_640261, EPI_ISL_640260, EPI_ISL_640223, EPI_ISL_640267, EPI_ISL_640222, EPI_ISL_640266, EPI_ISL_640221, EPI_ISL_640265, EPI_ISL_640220, EPI_ISL_640264, EPI_ISL_640227, EPI_ISL_640226, EPI_ISL_640225, EPI_ISL_640269, EPI_ISL_640224, EPI_ISL_640268, EPI_ISL_640229, EPI_ISL_640228, EPI_ISL_640270, EPI_ISL_640230, EPI_ISL_640272, EPI_ISL_640271, EPI_ISL_640234, EPI_ISL_640233, EPI_ISL_640232, EPI_ISL_640231, EPI_ISL_640238, EPI_ISL_640237, EPI_ISL_640236, EPI_ISL_640235, EPI_ISL_640239, EPI_ISL_640241, EPI_ISL_640240, EPI_ISL_640245, EPI_ISL_640244, EPI_ISL_640243, EPI_ISL_640242, EPI_ISL_640249, EPI_ISL_640248, EPI_ISL_640247, EPI_ISL_640246, EPI_ISL_640252, EPI_ISL_640251, EPI_ISL_640250, EPI_ISL_640256, EPI_ISL_640255, EPI_ISL_640254, EPI_ISL_640253, EPI_ISL_660540

**Supplemental Table 1:** Bioinformatic tools which were used for sequencing data analysis in this study.

| **Tool** | **Version** | **Source** |
| --- | --- | --- |
| ANNOVAR | 2018-04-16 | doi: 10.1093/nar/gkq603 |
| ARTIC pipeline | 1.0.0 | github.com/artic-network/artic-ncov2019, accession date: 22.04.2021 |
| ComplexHeatmap | 2.4.3 | doi: 10.1093/bioinformatics/btw313 |
| gggenes | 0.4.1 | CRAN.R-project.org/package=gggenes, accession date: 22.04.2021 |
| ggplot2 | 3.3.3 | ggplot2.tidyverse.org, accession date: 22.04.2021 |
| ggtree | 2.2.4 | doi: 10.1111/2041-210X.12628 |
| ggpubr | 0.4.0 | CRAN.R-project.org/package=ggpubr, accession date: 22.04.2021 |
| Guppy | 3.6.0 | nanoporetech.com, accession date: 22.04.2021 |
| MAFFT | 7.471 | doi: 10.1093/nar/gkf436 |
| Pangolin | 2.1.7 | github.com/cov-lineages/pangolin, accession date: 22.04.2021 |
| PHyML | 3.3.20200621 | doi: 10.1093/sysbio/syq010 |
| pycoQC | 2.5.0.17 | doi: 10.21105/joss.01236 |
| Rampart | 1.1.0 | github.com/artic-network/rampart, accession date: 22.04.2021 |

**Supplemental Table 2:** Variants which were identified by SARS-CoV-2 whole genome sequencing of 55 samples from COVID-19 patients. Only variants with a count >= 2 and <55 are shown. These variants were included into statistical analysis.

| **Nucleotide position** | **Count** | **Percentage  [%]** | **Gene^[a]^** | **Function^[a]^** | **Aminoacid Change^[a]^** |
| --- | --- | --- | --- | --- | --- |
| 1059 | 12 | 21.8 | ORF1a ORF1ab nsp2 | non-synonymous SNV | ORF1ab:YP_009724389.1:exon1:c.C794T:p.T265I ORF1a:YP_009725295.1:exon1:c.C794T:p.T265I nsp2:YP_009725298.1:exon1:c.C254T:p.T85I |
| 3276 | 3 | 5.5 | ORF1a ORF1ab nsp3 | non-synonymous SNV | ORF1ab:YP_009724389.1:exon1:c.C3011T:p.T1004 ORF1a:YP_009725295.1:exon1:c.C3011T:p.T1004I nsp3:YP_009742610.1:exon1:c.C557T:p.T186I |
| 3373 | 2 | 3.6 | ORF1a ORF1ab nsp3 | non-synonymous SNV | ORF1ab:YP_009724389.1:exon1:c.C3108A:p.D1036E ORF1a:YP_009725295.1:exon1:c.C3108A:p.D1036E nsp3:YP_009742610.1:exon1:c.C654A:p.D218E |
| 5842 | 2 | 3.6 | ORF1a ORF1ab nsp3 | synonymous SNV | ORF1ab:YP_009724389.1:exon1:c.C5577T:p.Y1859Y ORF1a:YP_009725295.1:exon1:c.C5577T:p.Y1859Y nsp3:YP_009742610.1:exon1:c.C3123T:p.Y1041Y |
| 7279 | 10 | 18.2 | ORF1a ORF1ab nsp3 | synonymous SNV | ORF1ab:YP_009724389.1:exon1:c.C7014T:p.F2338F ORF1a:YP_009725295.1:exon1:c.C7014T:p.F2338F nsp3:YP_009742610.1:exon1:c.C4560T:p.F1520F |
| 9559 | 2 | 3.6 | ORF1a ORF1ab nsp4 | synonymous SNV | ORF1ab:YP_009724389.1:exon1:c.C9294T:p.Y3098Y ORF1a:YP_009725295.1:exon1:c.C9294T:p.Y3098Y nsp4:YP_009725300.1:exon1:c.C1005T:p.Y335Y |
| 10323 | 2 | 3.6 | ORF1a ORF1ab nsp5 | non-synonymous SNV | ORF1ab:YP_009724389.1:exon1:c.A10058G:p.K3353R ORF1a:YP_009725295.1:exon1:c.A10058G:p.K3353R nsp5:YP_009725301.1:exon1:c.A269G:p.K90R |
| 12738 | 5 | 9.1 | ORF1a ORF1ab nsp9 | non-synonymous SNV | ORF1ab:YP_009724389.1:exon1:c.C12473T:p.T4158I ORF1a:YP_009725295.1:exon1:c.C12473T:p.T4158I nsp9:YP_009725305.1:exon1:c.C53T:p.T18I |
| 14772 | 17 | 30.9 | ORF1ab nsp12 | non-synonymous SNV | ORF1ab:YP_009724389.1:exon2:c.G14508T:p.Q4836H nsp12:YP_009725307.1:exon2:c.G1332T:p.Q444H |
| 15324 | 13 | 23.6 | ORF1ab nsp12 | synonymous SNV | ORF1ab:YP_009724389.1:exon2:c.C15060T:p.N5020N nsp12:YP_009725307.1:exon2:c.C1884T:p.N628N |
| 15380 | 4 | 7.3 | ORF1ab nsp12 | non-synonymous SNV | ORF1ab:YP_009724389.1:exon2:c.G15116T:p.S5039I nsp12:YP_009725307.1:exon2:c.G1940T:p.S647I |
| 16428 | 3 | 5.5 | ORF1ab nsp13 | synonymous SNV | nsp13:YP_009725308.1:exon1:c.C192T:p.Y64Y ORF1ab:YP_009724389.1:exon2:c.C16164T:p.Y5388Y |
| 22441 | 5 | 9.1 | S | synonymous SNV | S:YP_009724390.1:exon1:c.T879C:p.L293L |
| 25550 | 21 | 38.2 | ORF3a | non-synonymous SNV | ORF3a:YP_009724391.1:exon1:c.T158A:p.L53H |
| 25563 | 12 | 21.8 | ORF3a | nonsynonymous SNV | ORF3a:YP_009724391.1:exon1:c.G171T:p.Q57H |
| 25922 | 21 | 38.2 | ORF3a | nonsynonymous SNV | ORF3a:YP_009724391.1:exon1:c.G530T:p.S177I |
| 26530 | 21 | 38.2 | M | nonsynonymous SNV | M:YP_009724393.1:exon1:c.A8G:p.D3G |
| 28507 | 2 | 3.6 | N | synonymous SNV | N:YP_009724397.2:exon1:c.C234T:p.S78S |
| 28881 | 3 | 5.5 | N | non-synonymous SNV | N:YP_009724397.2:exon1:c.G608A:p.R203K |
| 28882 | 3 | 5.5 | N | synonymous SNV | N:YP_009724397.2:exon1:c.G609A:p.R203R |
| 28883 | 3 | 5.5 | N | nonsynonymous SNV | N:YP_009724397.2:exon1:c.G610C:p.G204R |
| 29031 | 2 | 3.6 | N | non-synonymous SNV | N:YP_009724397.2:exon1:c.A758C:p.E253A |
| 29485 | 2 | 3.6 | N | synonymous SNV | N:YP_009724397.2:exon1:c.C1212T:p.S404S |

[a] ANNOVAR Output

SNV, single nucleotide variation

**Supplemental Table 3:** Frequency of SARS-CoV-2 lineages. Whole genome sequencing was performed for 55 COVID-19 patients. The earliest description date in the Pango lineages data base is shown (Version 2021-01-16). Lineages B.1, B.1.126 and B.1.5 were significantly more prevalent than lineages B.1.1, B.1.322 and B.1.353 (Fisher’s exact test, p<0.05, respectively).

| **Lineage^[a]^** | **Number** | **Percentage [%]** | **Earliest Date^[b]^** |
| --- | --- | --- | --- |
| B.1 | 12 | 21.8 | 2020-01-24 |
| B.1.1 | 3 | 5.5 | 2020-01-08 |
| B.1.126 | 21 | 38.2 | 2020-05-05 |
| B.1.322 | 1 | 1.8 | n.a. ^[c]^ |
| B.1.353 | 2 | 3.6 | n.a. ^[c]^ |
| B.1.5 | 16 | 29.1 | n.a. ^[c]^ |

[a] pangoLEARN Version 2021-01-16
[b] Source: https://cov-lineages.org/lineages.html (access: 24.03.2021)
[c] Lineage has been reassigned in the mean time

**Supplemental Table 4:** Univariate regression analyses of COVID-19 patient characteristics.

A) Univariate regression analyses of binary COVID-19 patient characteristics by logistic regression analysis. The relationship of dichotomous COVID-19 patient characteristics as dependent variables and one independent parameter of patient characteristics listed in Table 1, SARS-CoV-2 genetic features or anti-SARS-CoV-2 antibodies as predictor was analysed.

B) Univariate regression analyses of quantitative COVID-19 patient characteristics as dependent variables and one independent parameter of patient characteristics listed in Table 1, SARS-CoV-2 genetic features or anti-SARS CoV-2 antibodies as predictor.

anti-S/N, SARS-CoV-2 antibodies against a mixture of the spike glycoprotein with the nucleocapsid; anti-S1 IgG, IgG antibodies to spike glycoprotein domain 1; anti-S2 IgG, IgG antibodies to spike glycoprotein domain 2; anti-N IgG, IgG antibodies to nucleocapsid

| **A) Univariate logistic regression** |  |  |  |  |  |
| --- | --- | --- | --- | --- | --- |
|  | **Coefficient** | **Std. Error** | **Odds ratio** | **95% CI** | **P Value** |
| Appetite loss |  |  |  |  |  |
| Blood type O | -1.396 | 0.657 | 0.248 | 0.068 - 0.898 | 0.0337 |
| anti-S/N IgG | 0.367 | 0.114 | 1.443 | 1.155 - 1.802 | 0.0012 |
| anti-S1 IgG | 0.174 | 0.082 | 1.190 | 1.013 - 1.398 | 0.0340 |
| anti-N IgG | 0.386 | 0.133 | 1.471 | 1.132 - 1.911 | 0.0038 |
| Overweight ^[a]^ | 1.250 | 0.632 | 3.492 | 1.012 – 12.052 | 0.0479 |
| Breathing difficulties |  |  |  |  |  |
| anti-S1 IgG | 0.341 | 0.115 | 1.407 | 1.124 - 1.761 | 0.0029 |
| anti-N IgG | 0.356 | 0.176 | 1.427 | 1.011 - 2.014 | 0.0431 |
| Bronchial secretions |  |  |  |  |  |
| Blood type A+ | 1.749 | 0.7372 | 5.750 | 1.356 - 24.389 | 0.0177 |
| Cough |  |  |  |  |  |
| Blood type A+ | 1.473 | 0.698 | 4.364 | 1.112 - 17.128 | 0.0347 |
| NSP12 Q444H | -1.887 | 0.692 | 0.152 | 0.035 – 0.551 | 0.0064 |
| ORF3a L53H | -1.366 | 0.619 | 0.255 | 0.072 – 0.833 | 0.0274 |
| ORF3a S177I | -1.764 | 0.628 | 0.171 | 0.045 – 0.578 | 0.0061 |
| M D3G | -1.764 | 0.628 | 0.171 | 0.045 – 0.578 | 0.0061 |
| Night sweat |  |  |  |  |  |
| Blood type A+ | 1.764 | 0.694 | 5.833 | 1.498 - 22.711 | 0.0110 |
| anti-S/N IgG | 0.400 | 0.143 | 1.492 | 1.127 - 1.976 | 0.0052 |
| anti-S/N IgM | 0.279 | 0.125 | 1.322 | 1.034 - 1.690 | 0.0260 |
| anti-N IgG | 0.287 | 0.139 | 1.333 | 1.016 - 1.749 | 0.0383 |
| Overweight ^[a]^ | 1.476 | 0.731 | 4.375 | 1.045 – 18.322 | 0.0434 |
| Oxygen need |  |  |  |  |  |
| anti-S/N IgM | 0.373 | 0.143 | 1.452 | 1.097 - 1.921 | 0.0091 |
| Cardiovascular disease | 2.862 | 1.185 | 17.500 | 1.716 – 178.441 | 0.0157 |
| Pneumonia |  |  |  |  |  |
| anti-S/N IgM | 0.310 | 0.144 | 1.362 | 1.027 - 1.808 | 0.0317 |
| Hospitalization |  |  |  |  |  |
| anti-S/N IgM | 0.357 | 0.135 | 1.430 | 1.097 - 1.863 | 0.0082 |
| anti-S1 IgG | 0.374 | 0.172 | 1.454 | 1.038 - 2.037 | 0.0296 |
| Cardiovascular disease | 3.219 | 1.171 | 25.000 | 2.518 – 248.190 | 0.0060 |
| Taste and smell disorders |  |  |  |  |  |
| Female sex | 1.299 | 0.642 | 3.667 | 1.042 - 12.904 | 0.0430 |
| NSP12 Q444H | 1.695 | 0.836 | 5.444 | 1.058 – 28.011 | 0.0426 |
| **B) Univariate regression analysis** |  |  |  |  |  |
|  | **Coefficient** | | **Std. Error** | | **P Value** |
| Hospitalisation duration |  | |  | |  |
| Anti-S/N IgM | 0.584 | | 0.132 | | 0.0001 |
| Anti-S1 IgG | 0.267 | | 0.107 | | 0.0150 |
| BMI | 0.173 | | 0.068 | | 0.0153 |
| Diabetes | 3.976 | | 1.224 | | 0.0022 |
| Female sex | -2.450 | | 0.836 | | 0.0052 |
| Tumour disease | 8.511 | | 1.366 | | <0.0001 |
| Vitamin D supplementation | 2.643 | | 1.300 | | 0.0479 |
| Symptom duration |  | |  | |  |
| anti-S1 IgG | 1.018 | | 0.403 | | 0.0152 |
| Chronical lung disease | 13.327 | | 4.075 | | 0.0021 |
| NSP9 T18I | 10.529 | | 5.298 | | 0.0530 |
| N E253A | 23.3 | | 7.693 | | 0.0041 |

[a] Overweight was characterised by BMI >25.


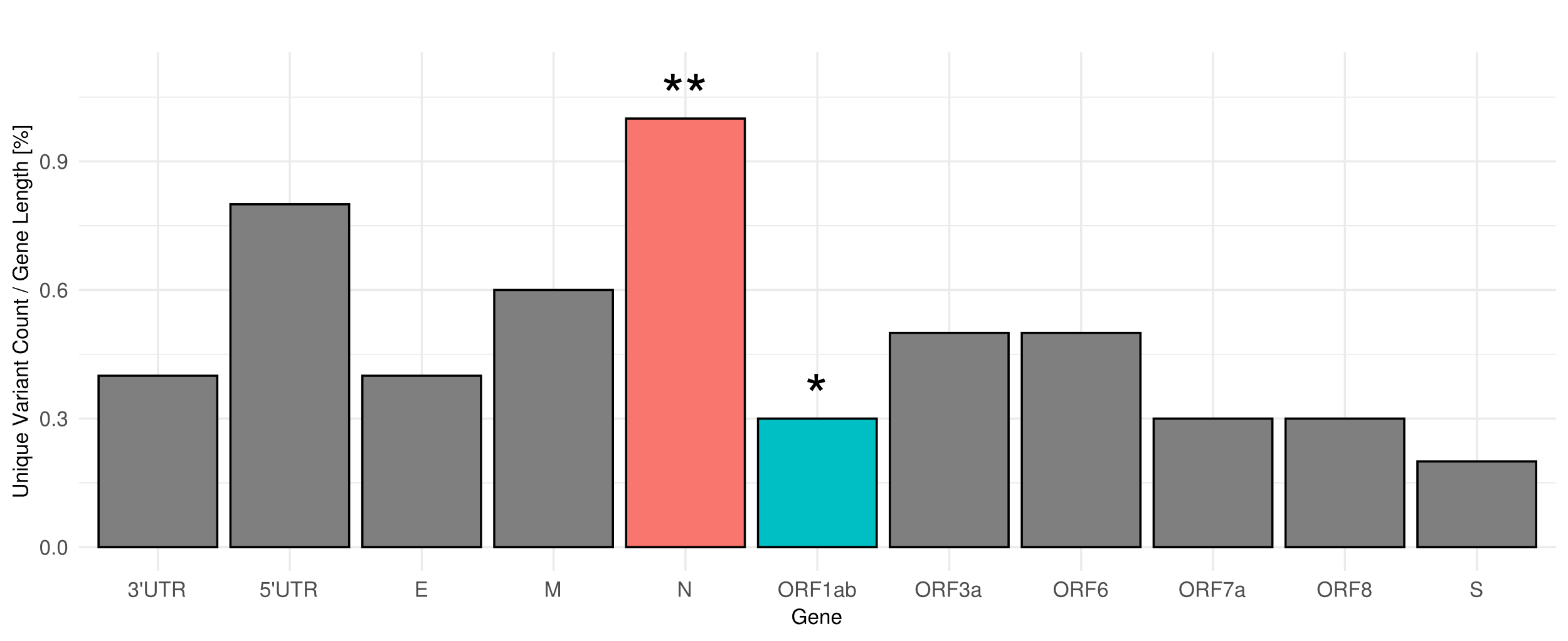


**Supplemental Figure 1:** Relative rate of unique variants in different genes of the SARS-CoV-2 genome normalized to their length. The N gene shows a significant higher variation rate (P=0.0096) compared to other regions by applying a general linearized model. ORF1ab shows a significant negative effect on the variation rate (P=0.04).
